# Synthesis of Tetrahydrohonokiol Derivates and Their Evaluation for Cytotoxic Activity against CCRF-CEM Leukemia, U251 Glioblastoma and HCT-116 Colon Cancer Cells

**DOI:** 10.3390/molecules19011223

**Published:** 2014-01-20

**Authors:** Marketa Bernaskova, Nadine Kretschmer, Wolfgang Schuehly, Antje Huefner, Robert Weis, Rudolf Bauer

**Affiliations:** 1 Department of Pharmaceutical Chemistry, Institute of Pharmaceutical Sciences, Universitätsplatz 1, University of Graz, 8010 Graz, Austria; E-Mails: marketa.bernaskova@uni-graz.at (M.B.); robert.weis@uni-graz.at (R.W.); 2 Department of Pharmacognosy, Institute of Pharmaceutical Sciences, Universitätsplatz 4, University of Graz, 8010 Graz, Austria; E-Mails: nadine.kretschmer@uni-graz.at (N.K.); wolfgang.schuehly@uni-graz.at (W.S.); rudolf.bauer@uni-graz.at (R.B.); 3 Institute of Zoologie, Universitätsplatz 2, University of Graz, 8010 Graz, Austria

**Keywords:** tetrahydrohonokiol derivatives, cytotoxicity, CCRF-CEM leukemia cells, U251 glioblastoma, HCT-116 colon cancer cells, *Magnolia officinalis*

## Abstract

Biphenyl neolignans such as honokiol and magnolol, which are the major active constituents of the Asian medicinal plant *Magnolia officinalis*, are known to exert a multitude of pharmacological and biological activities. Among these, cytotoxic and tumor growth inhibitory activity against various tumour cell lines are well-documented. To further elucidate the cytotoxic effects of honokiol derivatives, derivatizations were performed using tetrahydrohonokiol as a scaffold. The derivatizations comprised the introduction of functional groups, e.g., nitro and amino groups, as well as alkylation. This way, 18 derivatives, of which 13 were previously undescribed compounds, were evaluated against CCRF-CEM leukemia cells, U251 glioblastoma and HCT-116 colon cancer cells. The results revealed no significant cytotoxic effects in any of the three tested cell lines at a test concentration of 10 µM.

## 1. Introduction

Naturally occurring biphenyl neolignans such as honokiol (**1**) and the isomeric magnolol from the bark of Asian species of *Magnolia* (e.g., *M. officinalis* Rehd. et Wils. and *M. obovata* Thunb.) are considered the active principle in various multi-herb compositions of Chinese *materia medica*. Cortex Magnoliae officinalis (Hou Po) is listed in the Chinese Pharmacopoeia as a stomachic, an antihistamine, a sedative and used to treat cough and asthma, diarrhea and gastric ulcers [1,2]. Especially honokiol, 4'-*O*-methylhonokiol and further derivatives of honokiol are known for a great variety of biological and pharmacological activities [3]. Among these, anti-inflammatory activity through action on leukotriene and prostaglandin metabolism [4,5], CNS activity, identified as GABA_A_ receptor agonistic activity [6,7] and cannabinoid-2 receptor inverse agonistic activity [8] were reported and evaluated by the use of compound libraries.

Biphenyl-type compounds are considered to be privileged structures, *i.e.*, molecules that provide extraordinary binding properties toward proteinous targets. The biphenyl neolignan scaffold shows structural characteristics such as flexibility combined with partial rigidity and the presence of certain functional groups [9,10]. It provides an ideal scaffold for derivatizations that may be employed in structure activity relationship studies.

Cytotoxicity and tumour inhibition of honokiol derivatives have recently gained attention [11,12] despite the fact that ethnomedicinal data do not corroborate the use of *Magnolia* bark as a source of antineoplastic agents. Especially in most recent years, researchers have focused on biphenyl neolignans from *Magnolia* with cytotoxic and antitumour activity. Activity on various cancer cell lines was found to be caused by either direct cytotoxic effects or, e.g., by activation of apoptotic mechanisms [13,14,15,16]. Data about promising *in vivo* antineoplastic activity of honokiol are also available [17,18].

In a straightforward attempt to evaluate cytotoxic activity of derivatives of tetrahydrohonokiol (**2**), an array of 18 derivatives **2a**–**9b** was created. The rationale for the design of derivatizations was based on experiences with previous fruitful approaches on the honokiol scaffold applied to different targets [4,6,7]. The derivatizations in the presented work led to products with altered polarity through the introduction of alkyl groups as well as to products with nitrogen-containing pharmacophores in combination with polarity-altering alkyl groups. For an enhancement of overall molecular stability, tetrahydrohonokiol was used as starting compound because the allyl chains present in honokiol may undergo undesired chemical reactions. From our previous pharmacological results it was known that the tetrahydro derivatives of active biphenyl neolignans (e.g., honokiol, magnolol) did not exert significantly lower activities at the respective target than the non-hydrogenated parent compounds [7,8].

Natural product-guided compound libraries have been shown to be of great value for the exploration of lead structures and for the selection of new chemical entities [19]. The choice of tumour cell lines was done with regard to previous lead retrieval from botanical sources [20] and the clinical relevance of tumours associated with these cell lines as well as by considering multi-drug resistance in e.g. CCRF-CEM leukemia cells [21].

## 2. Results and Discussion

A series of 18 derivatives based on tetrahydrohonokiol as lead structure was synthesized using tetrahydrohonokiol (**2**) as starting material (Table 1). Compound **2** was used to provide overall higher molecular stability. The syntheses were aimed at providing compounds of different polarity by alkylation of one or two of the free hydroxy groups in **2** as well as to yield compounds with a nitrogen-containing pharmacophore at the position *ortho* to the phenolic OH-group of each ring. The latter compounds were further modified by variation of the polarity by reduction and *N*-acylation. This led to the nitro-substituted tetrahydrohonokiols **4a**,**b** and **7a**,**b**, amino-substituted tetrahydrohonokiols **5a**,**b** and **8a**,**b** and acetamido-substituted tetrahydro-honokiols **6a**,**b** and **9a**,**b**.

**Table 1:** Honokiol (**1**) and hydrogenated honokiols prepared from tetrahydrohonokiol (**2**). 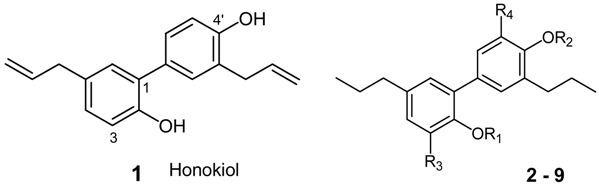

Compound	R_1_	R_2_	R_3_	R_4_
**2**	-H	-H	-H	-H
**2a**	-CH_3_	-H	-H	-H
**2b**	-H	-CH_3_	-H	-H
**2c**	-CH_3_	-CH_3_	-H	-H
**3a**	-C_2_H_5_	-H	-H	-H
**3b**	-H	-C_2_H_5_	-H	-H
**3c**	-C_2_H_5_	-C_2_H_5_	-H	-H
**4a**	-CH_3_	-H	-H	-NO_2_
**4b**	-H	-CH_3_	-NO_2_	-H
**5a**	-CH_3_	-H	-H	-NH_2_
**5b**	-H	-CH_3_	-NH_2_	-H
**6a**	-CH_3_	-H	-H	-NHCOCH_3_
**6b**	-H	-CH_3_	-NHCOCH_3_	-H
**7a**	-C_2_H_5_	-H	-H	-NO_2_
**7b**	-H	-C_2_H_5_	-NO_2_	-H
**8a**	-C_2_H_5_	-H	-H	-NH_2_
**8b**	-H	-C_2_H_5_	-NH_2_	-H
**9a**	-C_2_H_5_	-H	-H	-NHCOCH_3_
**9b**	-H	-C_2_H_5_	-NHCOCH_3_	-H

**Scheme 1:**
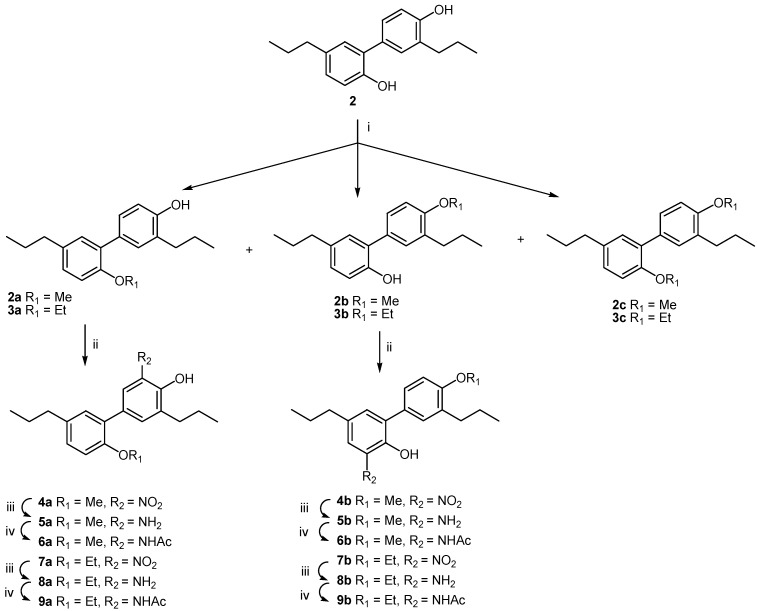
Synthesis of tetrahydrohonokiol derivatives **2a**–**9b**.

Starting from tetrahydrohonokiol (**2**), which is available from honokiol (**1**) [21,22], *O*-methylation and *O*-ethylation through the microwave procedure of Schuehly *et al.* [7] resulted in the monoalkylated key intermediates **2a+b** and **3a+b**, respectively, together with the respective byproduct **2c** or **3c** in moderate yield. Nitration in the position *ortho* to the free hydroxyl group according to Johnson *et al.* [23] resulted in **4a+b** and **7a+b** respectively, which were subsequently reduced to the corresponding amines **5a+b** and **8a+b** according to the literature procedure [24] and then *N*-acetylated with acetic anhydride in water [17] to yield **6a+b** and **9a+b**, respectively (Scheme 1).

*Reagents and Conditions*: (i) 1. KOH, 2. Me_2_SO_4_, MW irradiation or 1. KOH, 2. Et_2_SO_4_, MW irradiation; (ii) HNO_3_ (65%) EtOAc; (iii) SnCl_2_·2H_2_O, EtOH; (iv) Ac_2_O, H_2_O.

**Figure 1:**
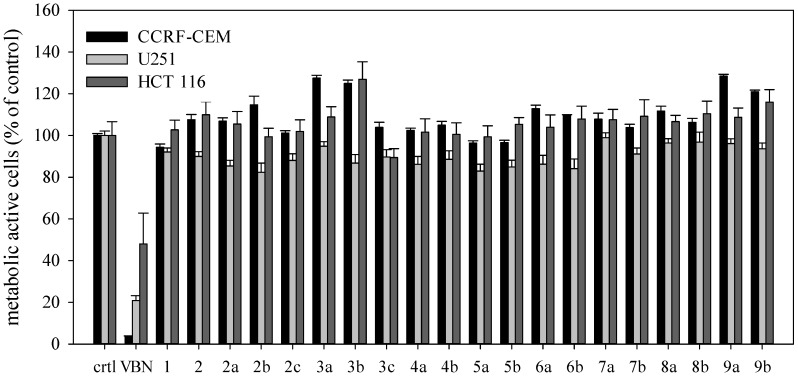
Effects of honokiol and tetrahydrohonokiol derivatives on cancer cell lines. Cells were incubated with the compound of interest for 72 h (n=6). Metabolic activity of the cells was measured using the XTT viability assay. Vehicle-treated cells (crtl, 0.5% DMSO) served as control, vinblastin (VBN) as positive control (0.01 µM). No cytotoxicity was found for any tetrahydrohonokiol derivative at 10 µM.

The cytotoxic/cytostatic activities against CCRF-CEM leukemia cells, U251 glioblastoma and HCT-116 colon cancer cells were evaluated for honokiol, tetrahydrohonokiol and the 18 derivatives thereof at a concentration of 10 µM. A higher test concentration was considered meaningless with regard to pharmacological relevance. The results indicated that neither honokiol nor any of the derivatives showed a significant cytotoxic effect at this concentration (Figure 1).

Most recently, data for the cytotoxic activity of honokiol and honokiol derivatives against various cancer cell lines, including leukemia Molt 4B cells [25], B-CLL cells [26], colon cancer cell lines such as HT-29 [27] and glioblastoma cells [28,29] were reported. Inhibitory effect on angiogenesis by honokiol [12,13,14] was also well reported and evaluated through a series of derivatives of honokiol [30]. Besides, the activities of synthetic neolignan analogs towards endothelial cells [31] were evaluated. Furthermore, the activity of honokiol against melanoma B16-F10 [32] and oral squamous [33] cells is documented. These results prompted us to seek for further evidence of cyctotoxic effects of yet unknown tetrahydrohonokiol derivatives. However, we could not confirm any cytotoxic effect using the three abovementioned cell lines. This may depend on the testing concentration. Reported IC_50_ values ranged from 25–30 µM in squamous cell lines [33], 30 µM in glioblastoma multiforme cells [29] and up to 60 µM in osteosarcoma cells [18]. However, these concentrations were used in the context of mechanistic studies in the respective cell systems. Herein, however, we aimed at synthesizing compounds with enhanced cytotoxic potential and, therefore, have set the test concentration to a pharmacologically meaningful threshold of 10 µM. From the chosen semi-synthetic approach, an enhancement of activity of the basic structure tetrahydrohonokiol would have been expected.

It appears that great expectations rely on the development of antineoplastic drugs based on honokiol or derivatives thereof. Honokiol was shown to be able to cross the blood brain barrier as well as to be orally bioavailable [16,17]. However, it may turn out that honokiol derivatives will not easily provide exploitable candidates for systemically applied antineoplastic drugs because of their incapability to survive the liver passage (first pass effect), which was demonstrated in metabolic studies in rats and human livers [34]. These studies indicated that the hepatic clearance of honokiol through glucuronidation and sulfation is very high. For further studies, including the drug safety of honokiol, evaluation of the metabolic stability and biotransformation of hydrogenated honokiols in comparison to the natural honokiol derivatives will also be necessary.

The derivatizations detailed out in this communication led to 13 new compounds that are described herein for the first time. Honokiol is known as a cytotoxic compound. We have chosen tetrahydrohonokiol because the hydrogenated products ascertained a greater stability as compared to the corresponding allyl derivatives and, therefore, greater reliability of the pharmacological results. The goal was to design derivatives with greater antitumour potential than honokiol through functionalization of the phenolic moieties. Given the clinical importance of the used tumour cell lines, any information about their susceptibility toward honokiol derivatives must be considered of interest. Hence, our finding, although unfruitful with regard to the effort to retrieve potential drug candidates for the use against tumours related to the tested cell lines, adds to the knowledge of cytotoxic properties of honokiol derivatives in general.

## 3. Experimental

### 3.1. General

Microwave reactions were carried out on a CEM Corp. Discover laboratory microwave equipped with an Explorer unit. Infrared spectra were recorded on a Bruker Alpha Platinum ATR spectrometer. ^1^H and ^13^C-NMR spectra were recorded on a Varian 400 MHz spectrometer (400 and 100 MHz, resp.) using chloroform-d as solvent and were referenced using TMS as internal standard. ESI-MS were recorded in ESI positive and negative on a LC Ultimate 3000 (Thermo, San José, CA, USA) with DAD detection in line with a Thermo Scientific LTQ XL mass spectrometer. Column: Knauer (Berlin, Germany) RP-18 (1.8 µm; 125 × 2.1 mm) with guard cartridge at a flowrate of 150 µL/min. For TLC analysis, precoated Si60 F_254_ plates (Merck, Darmstadt, Germany) were used. Detection was done by UV/254 nm and spraying with molybdatophosphoric acid and subsequent heating. Compound mixtures were separated by column chromatography using cyclohexane/AcOEt mixtures and through preparative HPLC (Varian Prepstar with Dynamax Rainin detector; column SepServ (Berlin, Germany) 250 × 21 mm, RP-18, 7 µm, flow rate 15 mL). Honokiol (purity > 98%) was purchased from APIChem Technology Co. (Hangzhou, China).

#### 3.1.1. General Procedure for Alkylation (Methylation and Ethylation, Respectively) of Tetrahydrohonokiol (**2**)

A microwave vial was charged with a stir bar, tetrahydrohonokiol (**2**, 1 mol eq.), KOH (3–4 mol eq.) and water/methanol (1:1, 4 mL). After stirring for 10 min the alkylation reagent (Et_2_SO_4_ or Me_2_SO_4_, 2 mmol eq.) was added. The reaction vessel was sealed and irradiated at 90 °C for 60 min. The reaction mixture was neutralized with aqueous HCl (1M) after cooling to room temperature and extracted with CH_2_Cl_2_ (3 × 10 mL). The organic phase was washed with brine (3 × 10 mL) and water (3 × 10 mL), dried over Na_2_SO_4_ and concentrated under reduced pressure. The obtained oil was purified by column chromatography on silica gel (cyclohexane/EtOAc = 9:1).

##### 3.1.1.1. 2-Methoxy-3',5-dipropylbiphenyl-2-ol (**2a**), 4'-methoxy-3',5-dipropylbiphenyl-2-ol (**2b**) and 2,4'-dimethoxy-3',5-dipropylbiphenyl-2-ol (**2c**)

Compound **2** (1.5 mmol) yielded 60 mg (18%) of **2a**, 39 mg (12%) of **2b** and 100 mg (31%) of **2c**. The NMR data of **2b** and **2c** agree with the literature [5]. Compound **2a**: colourless oil; IR (ATR, cm^−1^): 3428 (br, OH), 2956, 2929, 2869, 1607, 1508, 1463, 1264, 1237, 1118, 1027, 812; ^1^H-NMR (CDCl_3_): *δ* 1.04 (t, *J* = 7.3 Hz, 3H, H-3''), 1.08 (t, *J* = 7.7 Hz, 3H, H-3'''), 1.73 (sext, *J* = 7.3 Hz, 2H, H-2''), 1.76 (sext, *J* = 7.3 Hz, 2H, H-2'''), 2.64 (t, *J* = 7.3 Hz, 2H, H-1''), 2.70 (t, *J* = 7.7 Hz, 2H, H-1'''), 3.85 (s, 3H, OMe), 6.81 (d, *J* = 8.1 Hz, 1H, H-5'), 6.96 (d, *J* = 8.4 Hz, 1H, H-3), 7.16 (d, *J* = 8.4 Hz, 1H, H-4), 7.20 (s, 1H, H-6), 7.33 (d, *J* = 8.0 Hz, 1H, H-6'), 7.38 (s, 1H, H-2'); ^13^C-NMR (CDCl_3_): *δ* 13.8 (C-3''), 14.0 (C-3'''), 22.9 (C-2'''), 24.8 (C-2''), 32.1 (C-1'''), 37.2 (C-1''), 55.7 (OMe), 111.2 (C-3), 114.8 (C-5'), 127.7 (C-4), 127.9 (C-3'), 128.0 (C-6'), 130.3 (C-1), 130.8 (C-6), 130.9 (C-1'), 131.3 (C-2'), 134.9 (C-5), 152.5 (C-4'), 154.4 (C-2); ESI^+^ calcd for C_19_H_24_O_2_: [M]^+^ 284.18; found ESI-MS *m/z* (rel. int.): 284.21 [M]^+^ (100). This substance was reported by Rao and Davis without given spectroscopic information [35].

##### 3.1.1.2. 2-Ethoxy-3',5-dipropylbiphenyl-2-ol (**3a**), 4'-ethoxy-3',5-dipropylbiphenyl-2-ol (**3b**) and 2,4'-diethoxy-3',5-dipropylbiphenyl-2-ol (**3c**)

Compound **2** (610 mg, 2.26 mmol) yielded 148 mg (22%) of **3a**, 81 mg (12%) of **3b** and 214 mg (29%) of **3c** as colourless oils.

Compound **3a**: IR (ATR, cm^−1^): 3417 (br, OH), 2957, 2928, 2869, 1608, 1508, 1490, 1475, 1265, 1234, 1115, 1044, 814; ^1^H-NMR (CDCl_3_): *δ* 0.97 (t, *J* = 7.3 Hz, 3H, H-3''), 1.02 (t, *J* = 7.3 Hz, 3H, H-3'''), 1.35 (t, *J* = 7 Hz, 3H, CH_3_-OEt), 1.63 (sext, *J* = 7.3 Hz, 2H, H-2''), 1.71 (sext, *J* = 7.3 Hz, 2H, H-2'''), 2.58 (t, *J* = 7.3 Hz, 2H, H-1''), 2.64 (t, *J* = 7.3 Hz, 2H, H-1'''), 4.00 (q, *J* = 7.0 Hz, 2H, CH_2_-OEt), 6.79 (d, *J* = 8.4 Hz, 1H, H-5'), 6.88 (d, *J* = 8.4 Hz, 1H, H-3), 7.07 (dd, *J* = 8.4, 1.8 Hz, 1H, H-4), 7.13 (d, *J* = 1.8 Hz, 1H, H-6), 7.30 (dd, *J* = 8.4, 1.8 Hz, 1H, H-6'), 7.38 (d, *J* = 1.4 Hz, 1H, H-2'); ^13^C-NMR (CDCl_3_): *δ* 13.9 (C-3''), 14.1 (C-3'''), 14.9 (CH_3_-OEt), 22.9 (C-2'''), 24.8 (C-2''), 32.1 (C-1'''), 37.3 (C-1''), 64.2 (CH_2_-OEt), 112.8 (C-3), 114.7 (C-5'), 127.5 (C-3'), 127.6 (C-4), 128.1 (C-6'), 130.4 (C-1), 130.7 (C-6), 131.2 (C-1'), 131.5 (C-2'), 135.0 (C-5), 152.4 (C-4'), 153.8 (C-2); ESI^−^ calcd for C_20_H_26_O_2_: [M−H]^−^ 297.18; found ESI-MS *m/z* (rel. int.): 297.28 [M−H]^−^ (100).

Compound **3b**: 81 mg (12%). IR (ATR, cm^−1^): 3548 (br, OH), 2957, 2927, 2869, 1606, 1490, 1475, 1239, 1180, 1138, 1126, 1048, 811; ^1^H-NMR (CDCl_3_): *δ* 0.97 (t, *J* = 7.3 Hz, 3H, H-3''), 0.99 (t, *J* = 7.3 Hz, 3H, H-3'''), 1.46 (t, *J* = 7.0 Hz, 3H, CH_3_-OEt), 1.65 (sext, *J* = 7.3 Hz, 2H, H-2''), 1.67 (sext, *J* = 7.3 Hz, 2H, H-2'''), 2.56 (t, *J* = 7.3 Hz, 2H, H-1''), 2.65 (t, *J* = 7.3 Hz, 2H, H-1'''), 4.08 (q, *J* = 6.8 Hz, 2H, CH_2_-OEt), 6.90 (d, *J* = 8.8 Hz, 1H, H-3), 6.93 (d, *J* = 8.8 Hz, 1H, H-5'), 7.04 (s, 1H, H-6), 7.05 (d, *J* = 8 Hz, 1H, H-4), 7.24 (s, 1H, H-2'), 7.25 (d, *J* ~8 Hz, 1H, H-6'); ^13^C-NMR (CDCl_3_): *δ* 13.9 (C-3''), 14.1 (C-3'''), 14.9 (CH_3_-OEt), 22.9 (C-2'''), 24.8 (C-2''), 32.3 (C-1'''), 37.2 (C-1''), 63.6 (CH_2_-OEt), 111.7 (C-5'), 115.2 (C-3), 127.2 (C-6'), 127.8 (C-1), 128.5 (C-4), 128.7 (C-1'), 130.0 (C-6), 130.6 (C-2'), 132.3 (C-3'), 134.8 (C-5), 150.4 (C-2), 156.6 (C-4'); ESI^+^ calcd for C_20_H_26_O_2_: [M]^+^ 298.19; found ESI-MS *m/z* (rel. int.): 298.18 [M]^+^ (100).

Compound **3c**: IR (ATR, cm^−1^): 2957, 2928, 2869, 1607, 1492, 1475, 1235, 1134, 1045, 806; ^1^H-NMR (CDCl_3_): *δ* 0.97 (t, *J* = 7.3 Hz, 3H, H-3''), 0.99 (t, *J* = 7.3 Hz, 3H, H-3'''), 1.35 (t, *J* = 7.0 Hz, 3H, CH_3_-2-OEt), 1.45 (t, *J* = 7.0 Hz, 3H, CH_3_-4'-OEt), 1.66 (sext, *J* = 7.3 Hz, 2H, H-2''), 1.68 (sext, *J* = 7.3 Hz, 2H, H-2'''), 2.56 (t, *J* = 7.3 Hz, 2H, H-1''), 2.65 (t, *J* = 7.3 Hz, 2H, H-1'''), 4.00 (q, *J* = 7.0 Hz, 2H, CH_2_-2-OEt), 4.08 (q, *J* = 7.0 Hz, 2H, CH_2_-4'-OEt), 6.75 (d, *J* = 7.7 Hz, 1H, H-5'), 6.85 (d, *J* = 6.2 Hz, 1H, H-3), 7.06 (d, *J* = 8.4 Hz, 1H, H-4), 7.15 (s, 1H, H-6), 7.36 (s, 1H, H-6'), 7.40 (s, 1H, H-2'); ^13^C-NMR (CDCl_3_): *δ* 13.8 (C-3''), 14.2 (C-3'''), 14.9 (CH_3_-2-OEt), 15.0 (CH_3_-4'-OEt), 23.0 (C-2'''), 24.8 (C-2''), 32.4 (C-1'''), 37.3 (C-1''), 63.4 (CH_2_-4'-OEt), 64.1 (CH_2_-2-OEt), 110.7 (C-5'), 112.7 (C-3), 127.5 (C-4), 127.6 (C-6'), 130.3 (C-3'), 130.52, 130.54 (C-1 and C-1'), 130.8 (C-6), 131.2 (C-2'), 134.9 (C-5), 153.9 (C-2), 155.8 (C-4'); ESI^+^ calcd for C_22_H_30_O_2_: [M+H]^+^ 327.23; found ESI-MS *m/z* (rel. int.): 327.25 [M+H]^+^ (100).

#### 3.1.2. General Procedure for Nitration of Monoalkyl Tetrahydrohonokiols **2a**, **2b**, **3a** and **3b**

Aqueous nitric acid (65%, 10 mol eq.) was added under intense stirring within ca. 5 s to a solution of the resp. honokiol (1 mol eq.) in EtOAc at room temperature. The reaction mixture was stirred for 10 min and carefully neutralized with aqueous NaOH (2 M). The organic phase was separated, and the aqueous phase was extracted with EtOAc. The combined organic phases were washed with brine, dried over Na_2_SO_4_, and concentrated under reduced pressure. The obtained oil was purified by column chromatography on silica gel (cyclohexane/EtOAc = 49:1).

##### 3.1.2.1. 2-Methoxy-5'-nitro-3',5-dipropylbiphenyl-4'-ol (**4a**)

Compound **2a** (360 mg, 1.27 mmol) in EtOAc (10 mL) yielded 216 mg (52%) of **4a** as an orange oil. IR (ATR, cm^−1^): 3208 (br, OH), 2954, 2923, 2869, 1620, 1531 (NO_2_), 1504, 1461, 1318, 1244, 1135, 1028, 815, 668, 643; ^1^H-NMR (CDCl_3_): *δ* 0.98 (t, *J* = 7.3 Hz, 3H, H-3''), 1.25 (t, *J* = 7.3 Hz, 3H, H-3'''), 1.67 (sext, *J* = 7.3 Hz, 2H, H-2''), 1.73 (sext, *J* = 7.3 Hz, 2H, H-2'''), 2.59 (t, *J* = 7.3 Hz, 2H, H-1''), 2.76 (t, *J* = 7.3 Hz, 2H, H-1'''), 3.82 (s, 3H, OMe), 6.92 (d, *J* = 8.4 Hz, 1H, H-3), 7.12 (d, *J* = 2.2 Hz, 1H, H-6), 7.16 (dd, *J* = 8.4, 2.2 Hz, 1H, H-4), 7.67 (d, *J* = 1.8 Hz, 1H, H-2'), 8.15 (d, *J* = 1.8 Hz, 1H, H-6'), 10.98 (s, 1H, OH); ^13^C-NMR (CDCl_3_): *δ* 13.8 (C-3''), 13.9 (C-3'''), 22.5 (C-2'''), 24.8 (C-2''), 31.8 (C-1'''), 37.0 (C-1''), 55.6 (OMe), 111.2 (C-3), 122.9 (C-6'), 127.7 (C-1), 128.9 (C-4), 130.1 (C-1'), 130.3 (C-6), 132.8 (C-3'), 133.3 (C-5'), 135.2 (C-5), 139.2 (C-2'), 152.5 (C-4'), 154.4 (C-2); ESI^−^ calcd for C_19_H_23_NO_4_: [M−H]^−^ 328.15; found ESI-MS *m/z* (rel. int.): 328.26 [M−H]^−^ (100).

##### 3.1.2.2. 4'-Methoxy-3-nitro-3',5-dipropylbiphenyl-2-ol (**4b**)

Compound **2b** (435 mg, 1.46 mmol) yielded 370 mg (78%) of **4b** as an orange oil. IR (ATR, cm^−1^): 3168, 2958, 2930, 2869, 1607, 1537 (NO_2_), 1502, 1460, 1320 (sh, NO_2_), 1243, 1133, 1029, 813; ^1^H-NMR (CDCl_3_): 0.97 (t, *J* = 7.4 Hz, 3H, H-3''), 0.98 (t, *J* = 7.4 Hz, 3H, H-3'''), 1.65 (sext, 2H, H-2''), 1.67 (sext, *J* = 7.3 Hz, 2H, H-2'''), 2.60 (t, *J* = 7.4 Hz, 2H, H-1''), 2.64 (t, *J* = 7.4 Hz, 2H, H-1'''), 3.87 (s, 3H, OMe), 6.92 (d, *J* = 8.4 Hz, 1H, H-5'), 7.31 (d, *J* = 2.1 Hz, 1H, H-2'), 7.38 (dd, *J* = 8.3, 2.2 Hz, 1H, H-6'), 7.46 (d, *J* = 2.1 Hz, 1H, H-6), 7.88 (d, *J* = 2.1 Hz, 1H, H-4), 11.00 (s, 1H, OH); ^13^C-NMR (CDCl_3_): *δ* 13.6 (C-3''), 14.1 (C-3'''), 22.9 (C-2'''), 24.2 (C-2''), 32.3 (C-1'''), 36.8 (C-1''), 55.4 (OMe), 109.9 (C-5'), 122.5 (C-4), 127.8 (C-6'), 127.9 (C-1'), 130.8 (C-2'), 131.0 (C-3'), 132.7 (C-1), 133.7 (C-3), 134.2 (C-5), 138.8 (C-6), 151.0 (C-2), 157.3 (C-4'); ESI^−^ calcd for C_19_H_23_NO_4_: [M-H]^−^ 328.15; found ESI-MS *m/z* (rel. int.): 622.54 [2M−H]− (25), 328.26 [M−H]^−^ (100).

##### 3.1.2.3. 2-Ethoxy-5'-nitro-3',5-dipropylbiphenyl-4'-ol (**7a**)

Compound **3a** (128 mg, 0.430 mmol) yielded 129 mg (87%) of **7a** as a dark yellow oil. IR (ATR, cm^−1^): 3194 (br, OH), 2974, 2927, 2871, 1622, 1534 (NO_2_), 1502, 1463, 1320, 1241, 1134, 1044, 806, 668, 646; MS ^1^H-NMR (CDCl_3_): *δ* 0.94 (t, *J* = 7.3 Hz, 3H, H-3''), 0.99 (t, *J* = 7.3 Hz, 3H, H-3'''), 1.35 (t, *J* = 6.9 Hz, 3H, CH_3_-OEt), 1.66 (sext, *J* = 7.3 Hz, 2H, H-2 ''), 1.70 (sext, *J* = 7.3 Hz, 2H, H-2'''), 2.57 (t, *J* = 7.3 Hz, 2H, H-1''), 2.74 (t, *J* = 7.3 Hz, 2H, H-1'''), 4.03 (q, *J* = 6.9 Hz, 2H, CH_2_-OEt), 6.88 (d, *J* = 8.8 Hz, 1H, H-3), 7.12 (m, 2H, H-4, H-6), 7.71 (d, *J* = 1.8 Hz, 1H, H-2'), 8.18 (d, *J* = 1.8 Hz, 1H, H-6'); ^13^C-NMR (CDCl_3_): *δ* 13.8 (C-3''), 13.9 (C-3'''), 14.8 (CH_3_-OEt), 22.5 (C-2'''), 24.8 (C-2''), 31.8 (C-1'''), 37.1 (C-1''), 64.1 (CH_2_-OEt), 112.4 (C-3), 122.9 (C-6'), 127.7 (C-1), 128.9 (C-4), 130.2 (C-6), 130.3 (C-1'), 132.6 (C-3'), 133.3 (C-5'), 135.2 (C-5), 139.4 (C-2'), 152.4 (C-4'), 153.8 (C-2); ESI^+^ calcd for C_20_H_25_NO_4_: [M]^+^ 343.18; found ESI-MS *m/z* (rel. int.): 343.31 [M]^+^ (100).

##### 3.1.2.4. 4'-Ethoxy-3-nitro-3',5-dipropylbiphenyl-2-ol (**7b**)

Compound **3b** (61 mg, 0.20 mmol) gave 38 mg (55%) of **7b** as an orange oil. IR (ATR, cm^−1^): 3184 (OH), 2957, 2927, 2869, 1607, 1538 (NO_2_), 1502, 1459, 1320 (sh, NO_2_), 1242, 1132, 1044, 808; ^1^H-NMR (CDCl_3_): 0.97 (t, *J* = 7.3 Hz, 3H, H-3''), 0.98 (t, *J* = 7.3 Hz, 3H, H-3'''), 1.43 (t, *J* = 7.0 Hz, 3H, CH_3_-OEt), 1.64 (sext, *J* = 7.3 Hz, 2H, H-2''), 1.68 (sext, *J* = 7.3 Hz, 2H, H-2'''), 2.61 (t, *J* = 7.3 Hz, 2H, H-1''), 2.65 (t, *J* = 7.3 Hz, 2H, H-1'''), 4.09 (q, *J* = 7.0 Hz, 2H, CH_2_-OEt), 6.90 (d, *J* = 8.4 Hz, 1H, H-5'), 7.33 (d, *J* = 2.2 Hz, 1H, H-2'), 7.35 (dd, *J* = 8.4, 2.2 Hz, 1H, H-6'), 7.45 (d, *J* = 1.8 Hz, 1H, H-6), 7.88 (d, *J* = 2.2 Hz, 1H, H-4), 11.01 (s, 1H, OH); ^13^C-NMR (CDCl_3_): *δ* 13.6 (C-3''), 14.1 (C-3'''), 14.9 (CH_3_-OEt), 22.9 (C-2'''), 24.2 (C-2''), 32.4 (C-1'''), 36.8 (C-1''), 63.5 (CH_2_-OEt), 110.8 (C-5'), 122.4 (C-4), 127.6 (C-1'), 127.7 (C-6'), 130.8 (C-2'), 131.2 (C-3'), 132.8 (C-1), 133.7 (C-3), 134.2 (C-5), 138.8 (C-6), 151.1 (C-2), 156.8 (C-4'); ESI^−^ calcd for C_20_H_25_NO_4_: [M−H]^−^ 342.17; found ESI-MS *m/z* (rel. int.): 342.30 [M−H]^−^ (100).

#### 3.1.3. General Procedure for Reduction of Nitro Tetrahydrohonokiols **4a**, **4b**, **7a** and **7b**

SnCl_2_·2H_2_O (10 mol eq.) was added to a solution of the respective nitrohonokiol (1 mol eq.) in abs. EtOH. The reaction mixture was stirred for 48 h at room temperature, after that time an additional portion of SnCl_2_·2 H_2_O (10 mol eq.) was added. The reaction was stirred for another 24 h. The foamy precipitate resulting from the addition of aqueous NaHCO_3_ (1 M) was filtered with Celite^®^ and washed with EtOH. EtOH was distilled off (100 mbar) and the residue was extracted with CH_2_Cl_2_. The combined extracts were washed with aqueous NaHCO_3_ (1 M), water, dried over Na_2_SO_4_, and concentrated under reduced pressure. The residue was purified by PTLC or column chromatography (cyclohexane/EtOAc = 5:3).

##### 3.1.3.1. 5'-Amino-2-methoxy-3',5-dipropylbiphenyl-4'-ol (**5a**)

Compound **4a** (143 mg, 0.434 mmol) yielded 110 mg (85%) of **5a** as a brown oil. IR (ATR, cm^−1^): 3372 (br, NH), 3313 (br, NH; vbr OH), 2955, 2928, 2868, 1607 (NH), 1487, 1238, 1142, 1027, 807; ^1^H-NMR (CDCl_3_): *δ* 0.96 (t, *J* = 7.2 Hz, 3H, H-3''), 1.01 (t, *J* = 7.2 Hz, 3H, H-3'''), 1.64 (sext, *J* = 7.4 Hz, 2H, H-2''), 1.67 (sext, *J* = 7.3 Hz, 2H, H-2'''), 2.56 (t, *J* = 7.6 Hz, 2H, H-1''), 2.59 (t, *J* = 7.5 Hz, 2H, H-1'''), 3.78 (s, 3H, OMe), 6.81 (s, bs, 1H, H-2'), 6.87 (s, 1H, H-3), 6.88 (s, 1H, H-6'), 7.08 (s, 1H, H-4), 7.10 (s, 1H, H-6); ^13^C-NMR (CDCl_3_): *δ* 13.9 (C-3''), 14.1 (C-3'''), 22.0 (C-2'''), 24.8 (C-2''), 31.3 (C-1'''), 37.2 (C-1''), 55.7 (OMe), 111.1 (C-3), 117.3 (C-6'), 122.7 (C-2'), 127.6 (C-4), 128.0 (C-3'), 130.5 (C-1), 130.8 (C-6), 131.1 (C-1'), 133.3 (C-5'), 134.8 (C-5), 142.4 (C-4'), 154.5 (C-2); ESI^+^ calcd for C_19_H_25_NO_2_: [M+H]^+^ 300.20; found ESI-MS *m/z* (rel. int.): 300.22 [M+H]^+^ (100).

##### 3.1.3.2. 2-Amino-4'-methoxy-3',5-dipropylbiphenyl-2-ol (**5b**)

Compound **4b** (350 mg, 1.06 mmol) yielded 415 mg; 54 mg of this batch were purified by HPLC to give 26 mg of pure **5b** (63%). NMR data agree with Taferner *et al.* [7].

##### 3.1.3.3. 5'-Amino-2-ethoxy-3',5-dipropylbiphenyl-4'-ol (**8a**)

Compound **7a** (110 mg, 0.32 mmol) gave 12 mg (12%) of **8a** as a brown oil. IR (ATR, cm^−1^): 3364 (br, NH), 3316 (br, NH; vbr OH), 2956, 2927, 2669, 1607, 1488, 1235, 1142, 1042, 858, 804; ^1^H-NMR (CDCl_3_): *δ* 0.97 (t, *J* = 7.2 Hz, 3H, H-3''), 1.02 (t, *J* = 7.2 Hz, 3H, H-3'''), 1.26 (t, *J* = 6.7 Hz, 3H, CH_3_-OEt), 1.67 (sext, *J* = 7.2 Hz, 2H, H-2''), 1.70 (sext, *J* = 7.2 Hz, 2H, H-2'''), 2.58 (m, 4H, H-1'' and H-1'''), 4.01 (q, *J* = 6.9 Hz, 2H, CH_2_-OEt), 6.87 (m, 1H, H-3), 6.90 (m, 2H, H-2', H-6'), 7.06 (d, *J* = 7.7 Hz, 1H, H-4), 7.13 (s, 1H, H-6); ^13^C-NMR (CDCl_3_): *δ* 13.4 (C-3''), 14.0 (C-3'''), 14.8 (CH_3_-OEt), 22.9 (C-2'''), 24.7 (C-2''), 32.1 (C-1'''), 37.2 (C-1''), 53.4 (CH_2_-OEt), 112.8 (C-3), 117.2 (C-6'), 122.9 (C-2'), 127.4 (C-4), 128.0 (C-3'), 130.4 (C-1), 130.5 (C-1'), 130.7 (C-6), 134.9 (C-5), 134.9 (C-5'), 142.3 (C-4'), 153.8 (C-2); ESI^+^ calcd for C_20_H_27_NO_2_: [M+H]^+^ 314.21; found ESI-MS *m/z* (rel. int.): 314.33 [M+H]^+^ (100).

##### 3.1.3.4. 2-Amino-4'-ethoxy-3',5-dipropylbiphenyl-2-ol (**8b**)

Compound **7b** (37 mg, 0.11 mmol) gave 24 mg (71%) of **8b** as a brown oil. IR (ATR, cm^−1^): 3553, 3375 (br, OH), 2955, 2926, 2869, 1607 (NH), 1503, 1487, 1475, 1240, 1132, 1044, 807; ^1^H-NMR (CDCl_3_): *δ* 0.95 (t, *J* = 7.3 Hz, 3H, H-3''), 0.97 (t, *J* = 7.3 Hz, 3H, H-3'''), 1.49 (t, *J* = 7.0 Hz, 3H, CH_3_-OEt), 1.62 (sext, *J* = 7.3 Hz, 2H, H-2''), 1.67 (sext, *J* = 7.3 Hz, 2H, H-2'''), 2.49 (t, *J* = 7.3 Hz, 2H, H-1''), 2.53 (t, *J* = 7.3 Hz, 2H, H-1'''), 4.07 (q, *J* = 7.0 Hz, 2H, CH_2_-OEt), 5.30 (s, 1H, OH), 6.48 (d, *J* = 1.8 Hz, 1H, H-6), 6.56 (d, *J* = 1.8 Hz, 1H, H-4), 6.92 (d, *J* = 8.4 Hz, 1H, H-5'), 7.21-7.25 (m, 2H, H-2', H-6'); ^13^C-NMR (CDCl_3_): *δ* 13.9 (C-3''), 14.1 (C-3'''), 14.9 (CH_3_-OEt), 22.9 (C-2'''), 24.7 (C-2''), 32.3 (C-1'''), 37.5 (C-1''), 63.6 (CH_2_-OEt), 111.7 (C-5'), 115.2 (C-4), 119.8 (C-6), 127.1 (C-6'), 127.6 (C-1), 129.0 (C-1'), 130.5 (C-2'), 132.3 (C-3'), 134.2 (C-3), 135.0 (C-5), 138.4 (C-2), 156.5 (C-4'); ESI^+^ calcd for C_20_H_27_NO_2_: [M+H]^+^ 314.21; found ESI-MS *m/z* (rel. int.): 314.21 [M+H]^+^ (100).

#### 3.1.4. General Procedure for Acetylation of Amino Tetrahydrohonokiols **5a**, **5b**, **8a** and **8b**

In a 10 mL round-bottom flask the respective amino derivative (1 mol eq.) was suspended in water (0.3 mL), and acetic anhydride (4 mol eq.) was added. The flask was allowed to rotate in a water bath at 80 °C for 5 min. After cooling to room temperature, the reaction mixture was quenched with aqueous NaHCO_3_ (1 M) and extracted with CH_2_Cl_2_. The combined extracts were washed with aqueous NaHCO_3_ (1 M) and water, dried over Na_2_SO_4_, and concentrated under reduced pressure.

##### 3.1.4.1. 5'-Acetamido-2-methoxy-3',5-dipropylbiphenyl-4'-ol (**6a**)

Compound **5a** (23 mg, 0.077 mmol) yielded 20 mg (77%) of **6a** as a brown oil. IR (ATR, cm^−1^): 3288 (br, NH), 2957, 2929, 2869, 1636 (CO), 1550 (NH), 1481, 1240, 1143, 1027; ^1^H-NMR (CDCl_3_): *δ* 0.95 (t, *J* = 7.2 Hz, 3H, H-3''), 0.99 (t, *J* = 7.2 Hz, 3H, H-3'''), 1.64 (sext, *J* = 7.2 Hz, 2H, H-2'''), 1.68 (sext, *J* = 7.2 Hz, 2H, H-2''), 2.22 (s, 3H, CH_3_-acetyl), 2.56 (t, *J* = 7.2 Hz, 2H, H-1''), 2.70 (t, *J* = 7.2 Hz, 2H, H-1'''), 3.77 (s, 3H, OMe), 6.88 (d, *J* = 8.0 Hz, 1H, H-3), 6.97 (d, *J* = 1.3 Hz, 1H, H-6'), 7.08 (s, 1H, H-6), 7.09 (dd, *J* = ~8, 1.7 Hz, 1H, H-4), 7.19 (d, *J* = 1.2 Hz, 1H, H-2'), 7.61 (s, 1H, NH), 8.78 (s, 1H, OH); ^13^C-NMR (CDCl_3_): *δ* 13.8 (C-3''), 14.1 (C-3'''), 23.0 (C-2'''), 23.6 (CH_3_ acetyl), 24.8 (C-2''), 32.7 (C-1'''), 37.2 (C-1''), 55.7 (OMe), 111.2 (C-3), 121.1 (C-6'), 125.0 (C-5'), 128.0 (C-4), 129.1 (C-2'), 129.5 (C-1), 130.3 (C-1'), 130.7 (C-6), 133.1 (C-3'), 135.0 (C-5), 146.3 (C-4'), 154.4 (C-2), 170.6 (CO acetyl); ESI^+^ calcd for C_21_H_27_NO_3_: [M+H]^+^ 342.21; found ESI-MS *m/z* (rel. int.): 342.27 [M+H]^+^ (100); 682.76 [2M]+ (54).

##### 3.1.4.2. 3-Acetamido-4'-methoxy-3',5-dipropylbiphenyl-2-ol (**6b**)

Compound **5b** (20 mg, 0.670 mmol) yielded 10 mg (45%) of **6b** as a brown oil. IR (ATR, cm^−1^): 3287 (br, NH), 2957, 2929, 2869, 1637 (CO), 1607, 1539 (NH), 1501, 1241, 1139, 1030, 812; ^1^H-NMR (CDCl_3_): *δ* 0.94 (t, *J* = 7.3 Hz, 3H, H-3''), 0.97 (t, *J* = 7.3 Hz, 3H, H-3'''), 1.58-1.68 (m, 4H, H-2'', H-2'''), 2.23 (s, 3H, CH_3-_acetyl), 2.52 (t, *J* = 7.3 Hz, 2H, H-1''), 2.63 (t, *J* = 7.3 Hz, 2H, H-1'''), 3.86 (s, 3H, OMe), 6.88 (d, *J* = 1.5 Hz, 1H, H-6), 6.92 (d, *J* = 8.4 Hz, 1H, H-5'), 7.01 (bs, 1H, OH), 7.25 (s, due to overlap with solvent, 1H, H-2'), 7.30 (dd, *J* = 8.2, 2.1 Hz, 1H, H-6'), 7.40 (d, *J* = 1.5 Hz, 1H, H-4), 7.70 (s, 1H, NH); ^13^C-NMR (CDCl_3_): *δ* 13.8 (C-3''), 14.2 (C-3'''), 22.9 (C-2'''), 24.2 (CH_3_ acetyl), 24.7 (C-2''), 32.3 (C-1'''), 37.3 (C-1''), 55.4 (CH_3_-OMe), 110.4 (C-5'), 120.1 (C-4), 125.9 (C-3), 126.6 (C-6), 127.5 (C-6'), 129.4 (C-1'), 130.0 (C-1), 130.8 (C-2'), 131.8 (C-3'), 134.8 (C-5), 141.7 (C-2), 157.0 (C-4'), 169.5 (CO acetyl); ESI^+^ calcd for C_21_H_27_NO_3_: [M+H]^+^ 342.21; found ESI-MS *m/z* (rel. int.): 342.23 [M+H]^+^ (100); 683.04 [2M+H]+ (46).

##### 3.1.4.3. 5'-Acetamido-2-ethoxy-3',5-dipropylbiphenyl-4'-ol (**9a**)

Compound **8a** (15 mg, 0.048 mmol) gave 8 mg (44%) of **9a** as a brown oil. IR (ATR, cm^−1^): 3290 (br, NH), 2957, 2927, 2869, 1637 (CO), 1540 (NH), 1476, 1237, 1143, 1041, 873, 805; ^1^H-NMR (CDCl_3_): *δ* 0.95 (t, *J* = 7.2 Hz, 3H, H-3''), 0.98 (t, *J* = 7.2 Hz, 3H, H-3'''), 1.26 (t, *J* = 6.7 Hz, 2H, CH_3_-OEt), 1.62 (sext, *J* = 7.2 Hz, 2H, H-2''), 1.69 (sext, *J* = 7.2 Hz, 2H, H-2'''), 2.23 (s, 3H, CH_3_-acetyl), 2.57 (t, *J* = 7.2 Hz, 2H, H-1''), 2.70 (t, *J* = 7.2 Hz, 2H, H-1'''), 3.99 (q, *J* = 6.9 Hz, 2H, CH_2_-OEt), 6.87 (d, *J* = 8.1 Hz, 1H, H-3), 6.96 (d, *J* = 1.8 Hz, 1H, H-6'), 7.06 (d, *J* = 8.1 Hz, 1H, H-4), 7.07 (s, 1H, H-6), 7.27 (s, 1H, H-2'); ^13^C-NMR (CDCl_3_): *δ* 13.9 (C-3''), 14.1 (C-3'''), 14.9 (CH_3_-OEt), 23.0 (C-2'''), 23.7 (CH_3_ acetyl), 24.78 (C-2''), 32.7 (C-1'''), 37.2 (C-1''), 64.3 (CH_2_-OEt), 112.9 (C-3), 120.9 (C-6'), 124.9 (C-5'), 127.9 (C-4), 129.4 (C-2'), 129.7 (C-1), 130.5 (C-1'), 130.6 (C-6), 132.9 (C-3'), 135.1 (C-5), 146.2 (C-4'), 153.8 (C-2), 170.5 (CO acetyl); ESI^+^ calcd for C_22_H_29_NO_3_: [M+H]^+^ 356.22; found ESI-MS *m/z* (rel. int.): 356.26 [M+H]^+^ (100), 710.99 [2M+H]+ (61).

##### 3.1.4.4. 3-Acetamido-4'-ethoxy-3',5-dipropylbiphenyl-2-ol (**9b**)

Compound **8b** (13 mg, 0.042 mmol) yielded 13 mg (90%) of **9b** as a brown oil. IR (ATR, cm^−1^): 3294 (br, OH), 2958, 2928, 2870, 1639 (CO), 1537 (NH), 1502, 1475, 1240, 1138, 1044, 907, 809; ^1^H-NMR (CDCl_3_): *δ* 0.94 (t, *J* = 7.3 Hz, 3H, H-3''), 0.97 (t, *J* = 7.3 Hz, 3H, H-3'''), 1.42 (t, *J* = 7.0 Hz, 3H, CH_3_-OEt), 1.63 (sext, *J* = 7.3 Hz, 2H, H-2''), 1.66 (sext, *J* = 7.3 Hz, 2H, H-2'''), 2.23 (s, 3H, CH_3_-acetyl), 2.53 (t, *J* = 7.3 Hz, 2H, H-1''), 2.63 (t, *J* = 7.3 Hz, 2H, H-1'''), 4.07 (q, *J* = 7.0 Hz, 2H, CH_2_-OEt), 6.88 (d, *J* = 1.8 Hz, 1H, H-6), 6.92 (d, *J* = 8.4 Hz, 1H, H-5'), 7.24-7.29 (m, 2H, H-2', H-6'), 7.45 (d, *J* = 1.5 Hz, 1H, H-4), 7.70 (bs, 1H, NH); ^13^C-NMR (CDCl_3_): *δ* 13.8 (C-3''), 14.1 (C-3'''), 14.9 (CH_3_-OEt), 22.9 (C-2'''), 24.3 (CH_3_ acetyl), 24.7 (C-2''), 32.4 (C-1'''), 37.4 (C-1''), 63.6 (CH_2_-OEt), 111.4 (C-5'), 120.0 (C-4), 125.9 (C-3), 126.5 (C-6), 127.4 (C-6'), 129.1 (C-1'), 129.9 (C-1), 130.7 (C-2'), 131.7 (C-3'), 134.8 (C-5), 141.5 (C-2), 156.5 (C-4'), 169.3 (CO acetyl); ESI^+^ calcd for C_22_H_29_NO_3_: [M+H]^+^ 356.22; found ESI-MS *m/z* (rel. int.): 356.29 [M+H]^+^ (95), 710.92 [2M+H]+ (100).

### 3.2. Cell Culture

Leukemia cells CCRF-CEM were cultured in RPMI 1640 medium (Gibco, Life Technologies Corporation, Vienna, Austria) supplemented with 2 mM glutamine (Sigma, Saint Louis, MO, USA), 10% heat-inactivated fetal bovine serum (FBS, PAA laboratories, Austria) and 1% Pen/Strep (PAA Laboratories, Pasching, Austria). Glioblastoma U251 and colon cancer HCT 116 cells were cultured in Dulbecco’s modified Eagle medium (DMEM, Gibco), 2 mM glutamine, 10% FBS and 1% Pen/Strep. All cells were kept at 37 °C in a humidified 5% CO_2_ atmosphere. 

### 3.3. XTT Assay

Honokiol derivatives were dissolved in DMSO and diluted with steril water. Cell proliferation kit II (XTT) (Cat. No 11465015001) was obtained from Roche Diagnostics (Mannheim, Germany). Aliquots (100 µL) of 5 × 10^4^ cells/well in case of U251 and HCT 116 cells were seeded in 96-well plates (flat bottom) and grown overnight before adding the compounds. 1 × 10^5^ cells/well (100 µL) of CCRF-CEM cells were seeded into 96-well plates and derivatives were added immediately. Control cells were treated with 0.5% DMSO (final DMSO concentration during the assay) which did not affect the cells. All cells were incubated with the compounds for 72 h at 37 °C/5% CO_2_ before XTT solution was added. XTT solution consisted of a XTT labelling reagent and an electron-coupling reagent. XTT is a yellow tetrazolium salt (sodium 3-[1-(phenylaminocarbonyl)-3,4-tetrazolium]-bis (4-methoxy-6-nitro)benzene sulfonic acid hydrate) that is cleaved by metabolic active cells into an orange formazan dye. This color change only occurs in viable cells and can be directly quantified using a scanning multiwell spectrophotometer [36]. Numbers of viable cells were determined with the following formula and expressed as percentage of control: (absorbance of treated cells/absorbance of untreated cells) × 100.

## 4. Conclusions

The present study aimed at both corroborating existing findings on the cytotoxic and anti-cancer activity of biphenyl-type neolignans and providing compounds that exert promising cytotoxic activity. Based on a rational access, we have used a scaffold that in some other pharmacological tests already turned out to be fruitful [5,7]. The choice of cancer cell lines and the test concentration of 10 µM was carefully considered in order to allow a most stringent approach. However, we cannot confirm from our results the recently quite frequent reports on the promising anti-cancer and cytotoxic activity of honokiol derivatives in our cell lines. Nevertheless we feel that the results are worth communicating and we hope that they may stimulate the discussion about honokiol as an anti-cancer drug lead.

## Supplementary Materials

Supplementary materials can be accessed at: http://www.mdpi.com/1420-3049/19/1/1223/s1.
