# Supplementary material for: Synthesis of Tetrahydrohonokiol Derivates and Their Evaluation for Cytotoxic Activity against CCRF-CEM Leukemia, U251 Glioblastoma and HCT-116 Colon Cancer Cells
Source: Molecules. 2014 Jan 20;19(1):1223–37. doi: 10.3390/molecules19011223 (PMC6270748; doi:10.3390/molecules19011223)

Supplementary Materials

^{1}H-NMR spectra ($CDCl_3$, 400 MHz) of the new compounds 3a–5a and 6a–9b

Figure S1. ^{1}H-NMR spectrum ($CDCl_3$, 400 MHz) of **3a**.

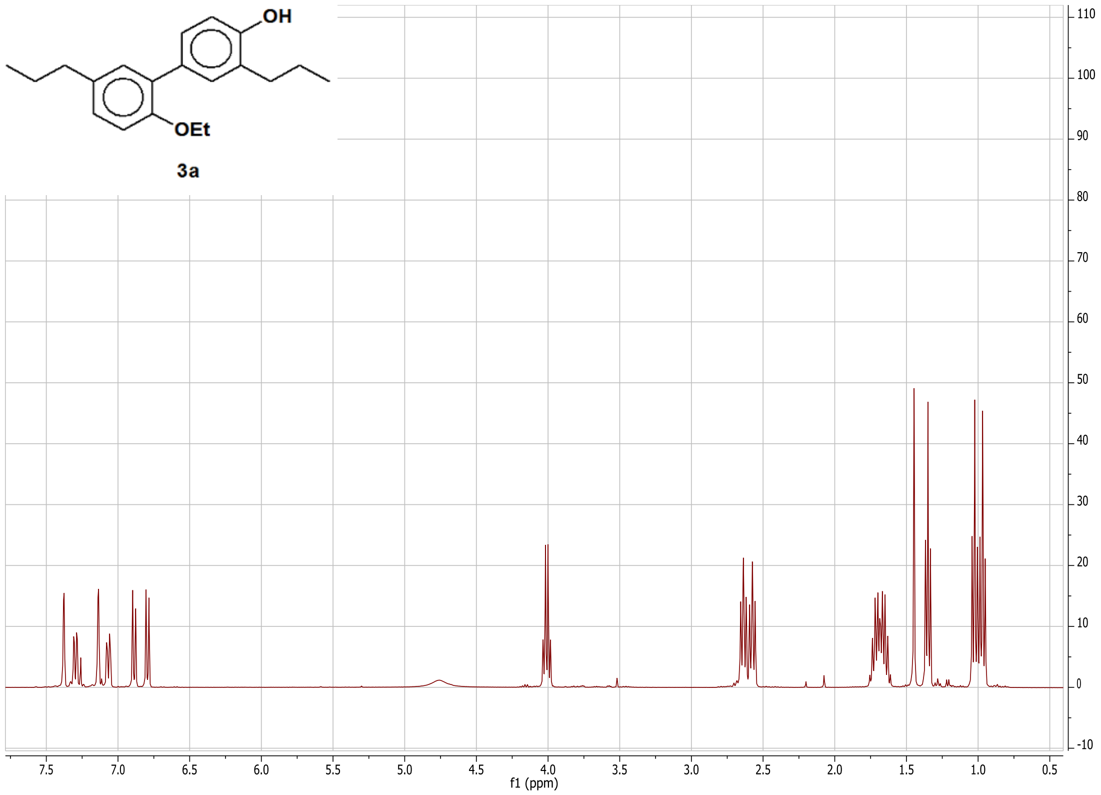

Figure S2. ^{1}H-NMR spectrum ($CDCl_3$, 400 MHz) of **3b**.

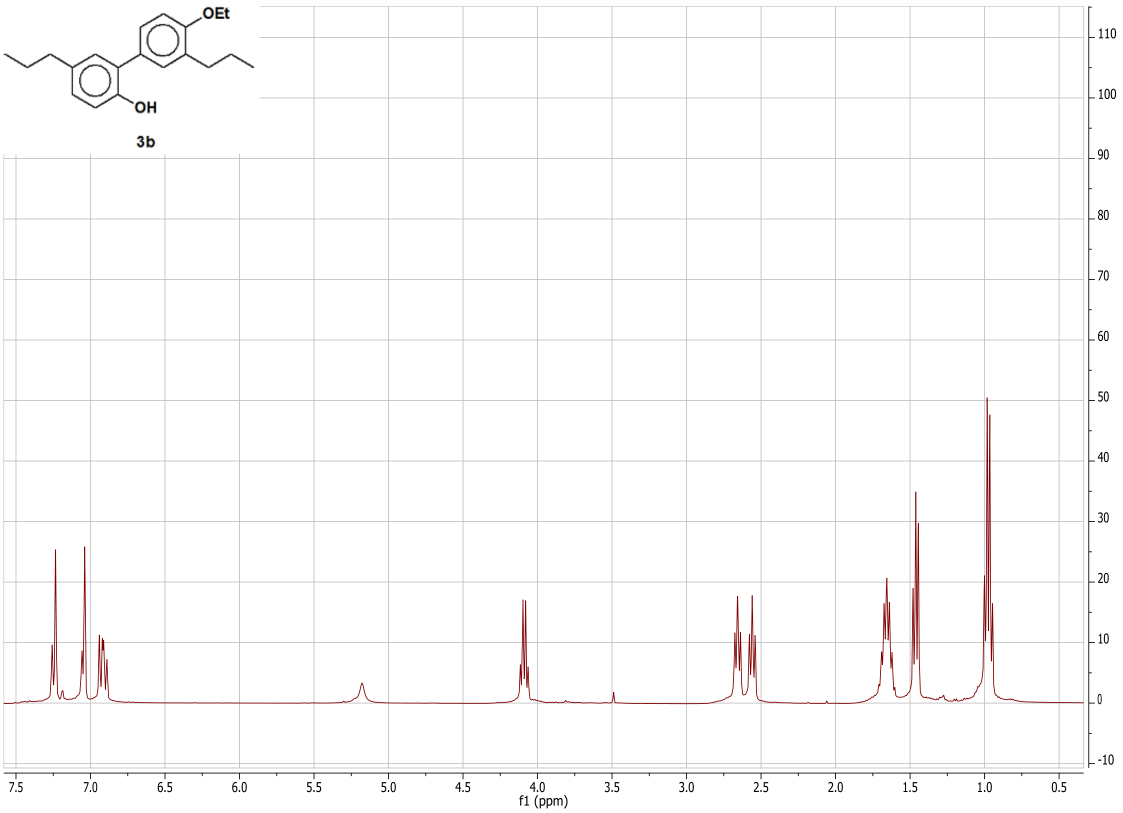

Figure S3. ^{1}H-NMR spectrum ($CDCl_3$, 400 MHz) of **3c**.

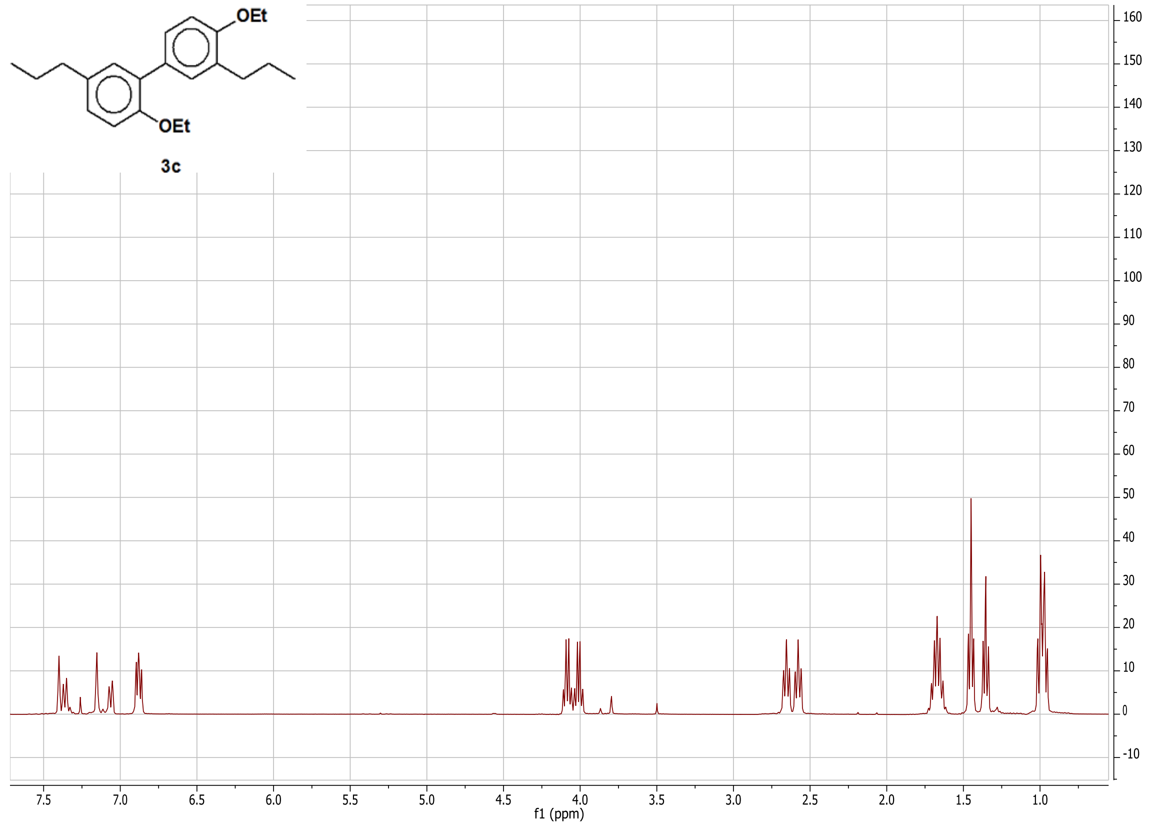

Figure S4. ^{1}H-NMR spectrum ($CDCl_3$, 400 MHz) of **4a**.

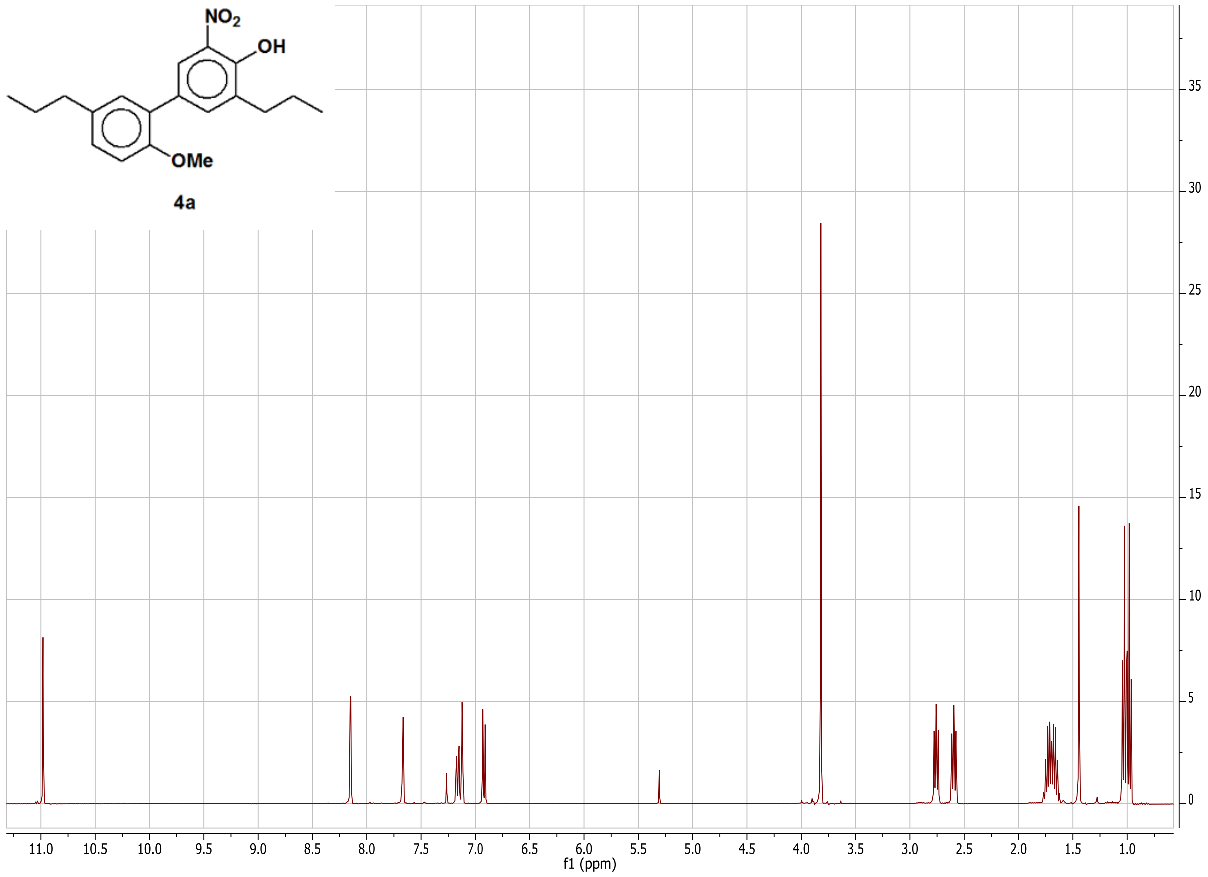

Figure S5. ^{1}H-NMR spectrum ($CDCl_3$, 400 MHz) of **4b**.

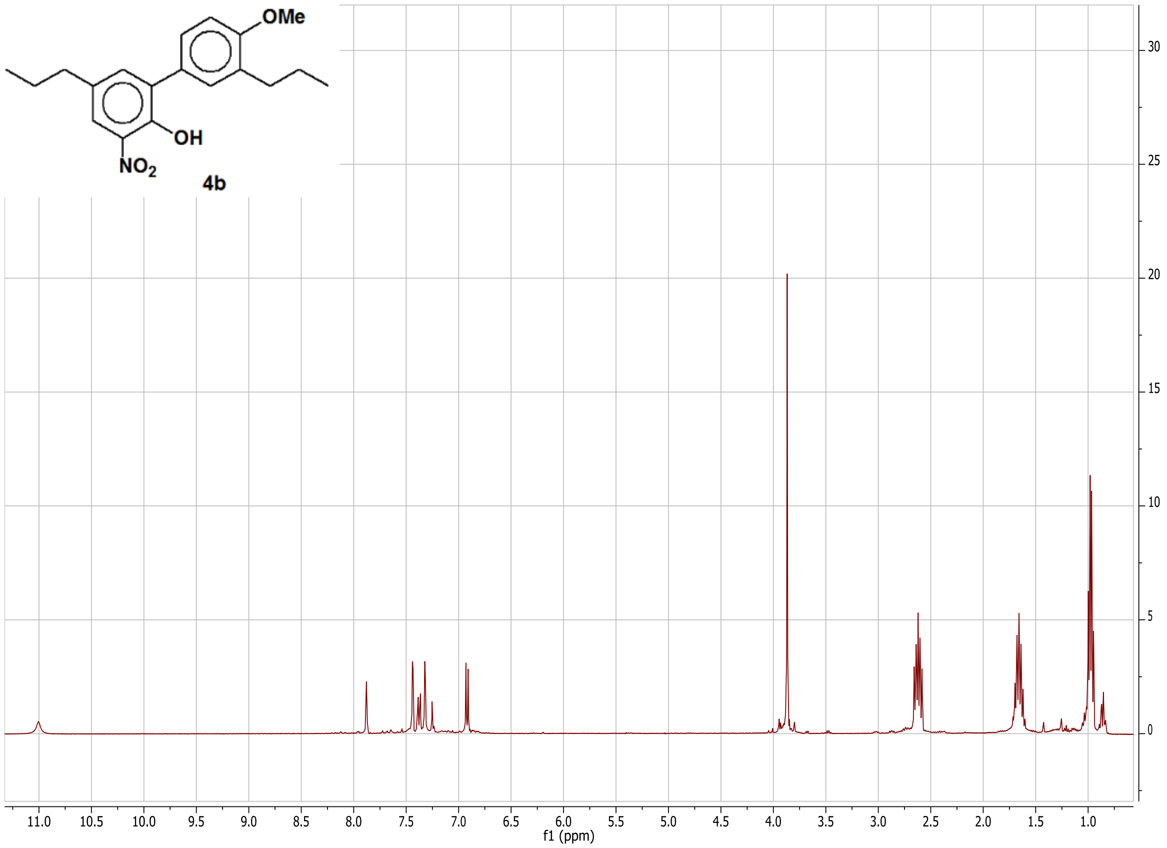

Figure S6. ^{1}H-NMR spectrum ($CDCl_3$, 400 MHz) of **5a**.

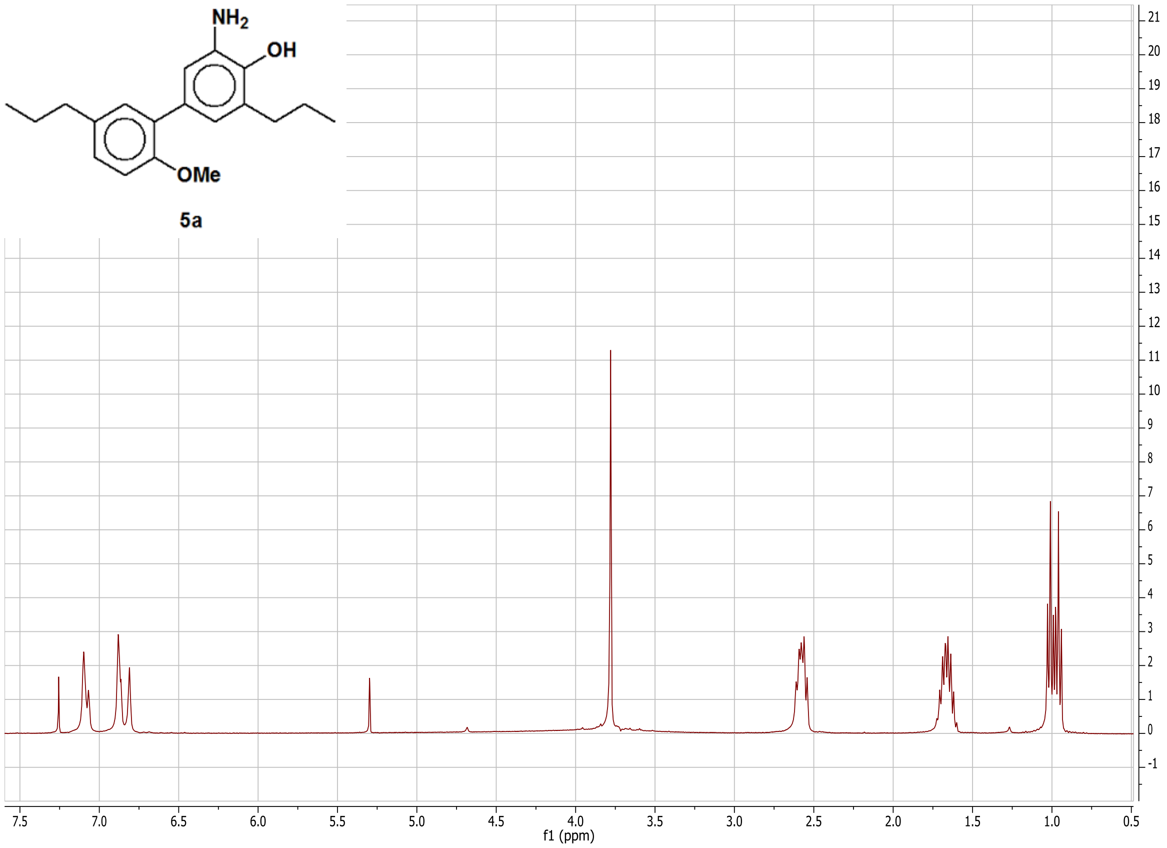

Figure S7. ^{1}H-NMR spectrum ($CDCl_3$, 400 MHz) of **6a**.

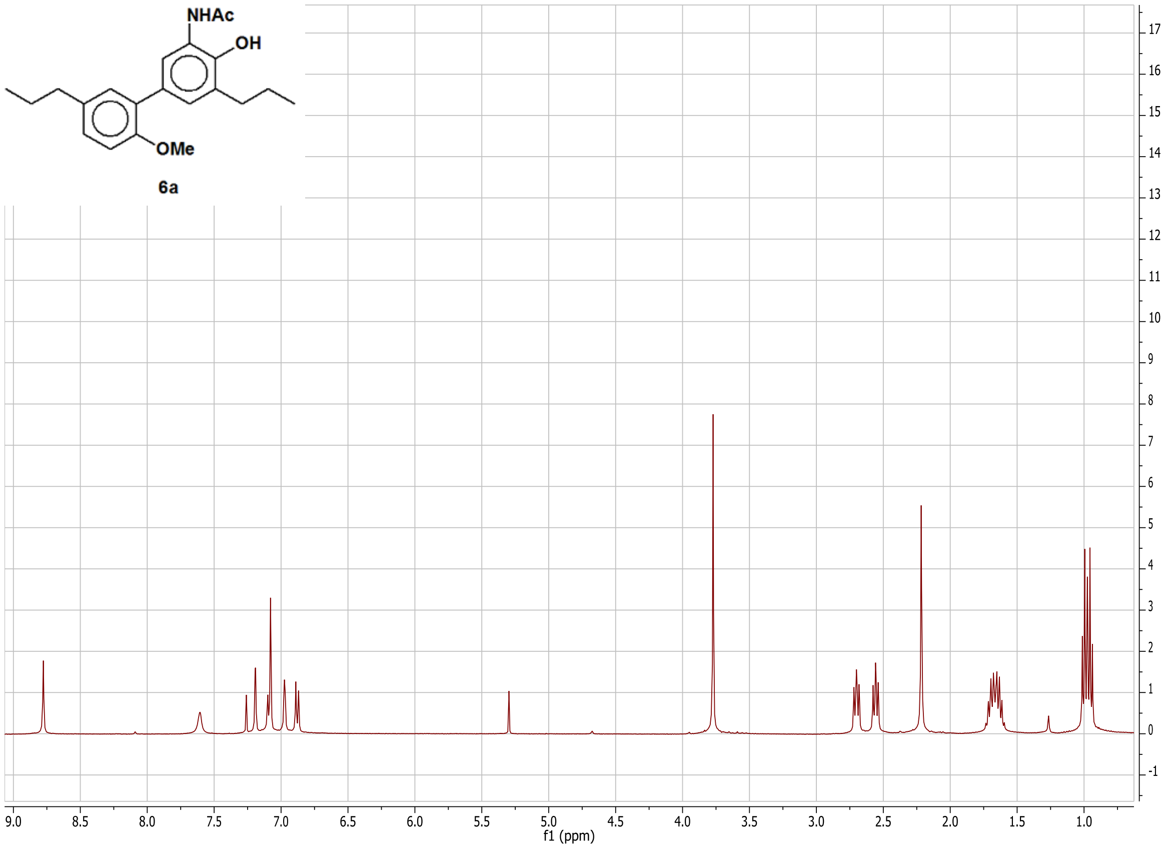

Figure S8. ^{1}H-NMR spectrum ($CDCl_3$, 400 MHz) of **6b**.

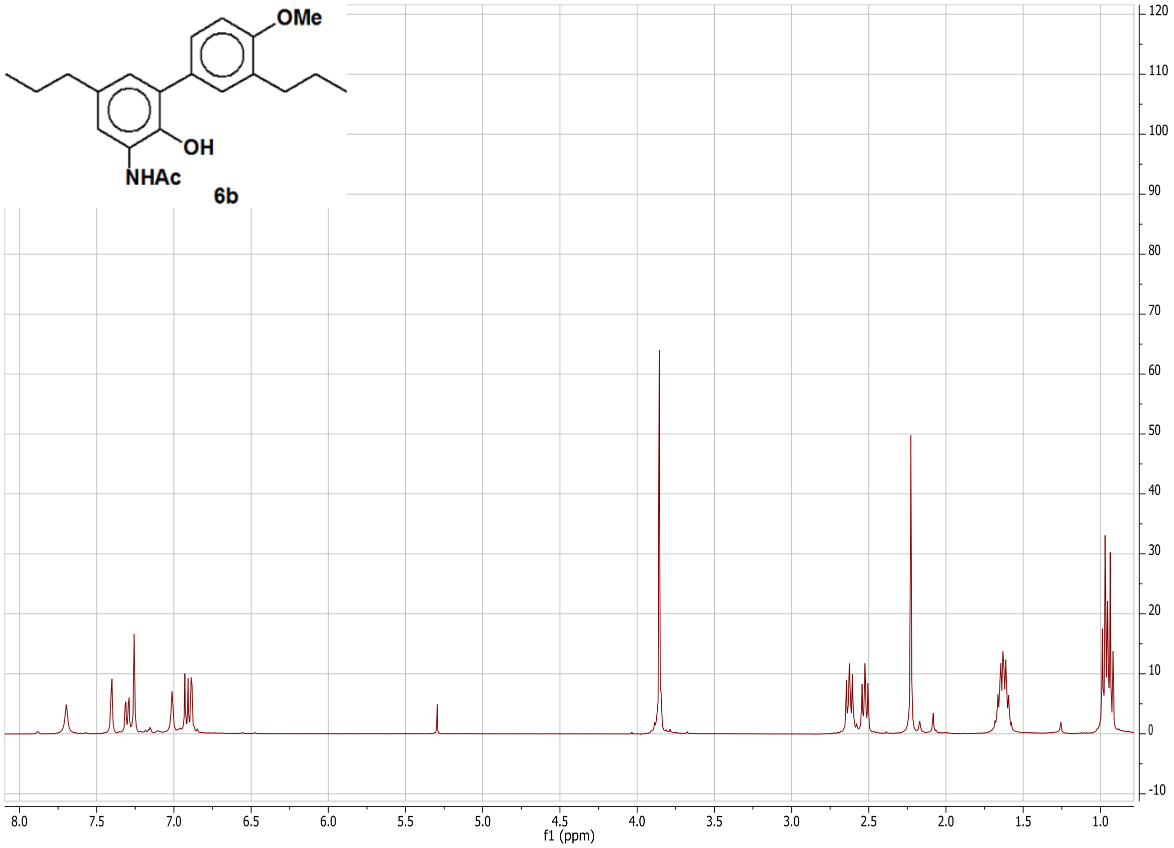

Figure S9. ^{1}H-NMR spectrum ($CDCl_3$, 400 MHz) of **7a**.

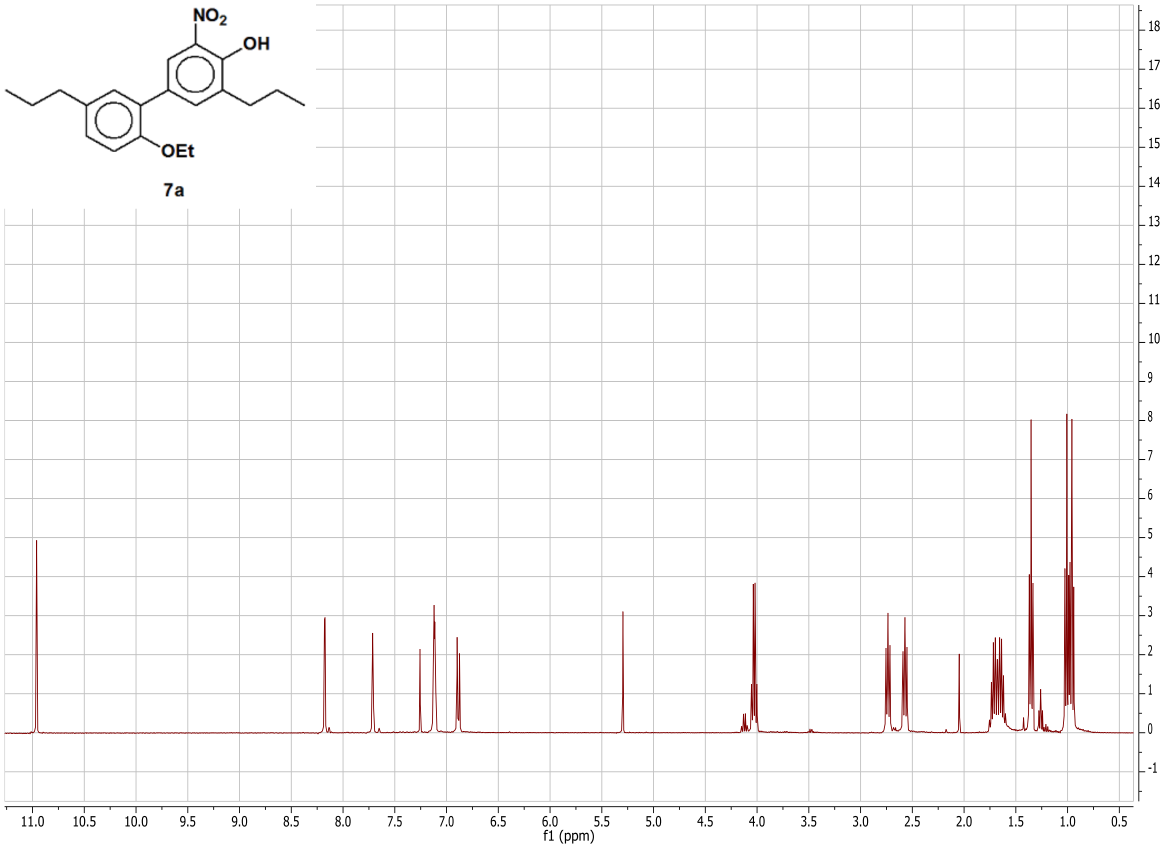

Figure S10. ^{1}H-NMR spectrum ($CDCl_3$, 400 MHz) of **7b**.

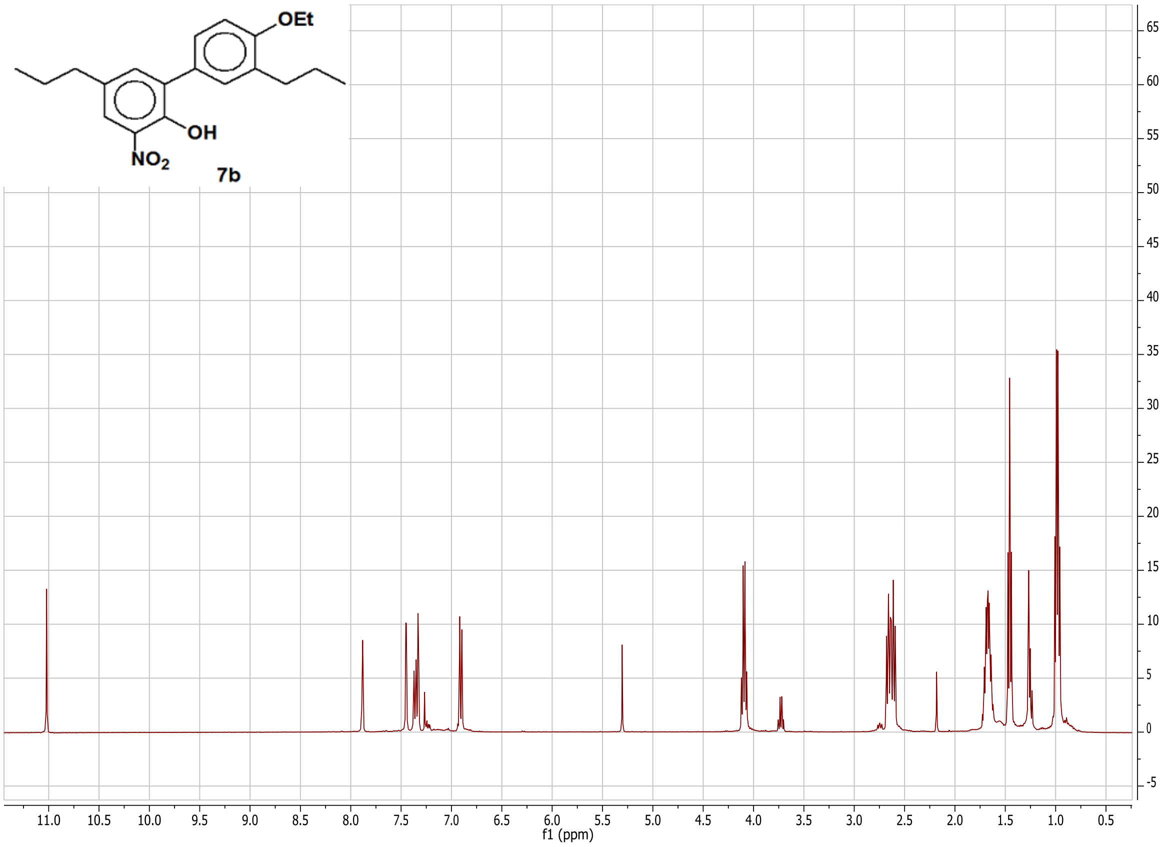

Figure S11. ^{1}H-NMR spectrum ($CDCl_3$, 400 MHz) of **8a**.

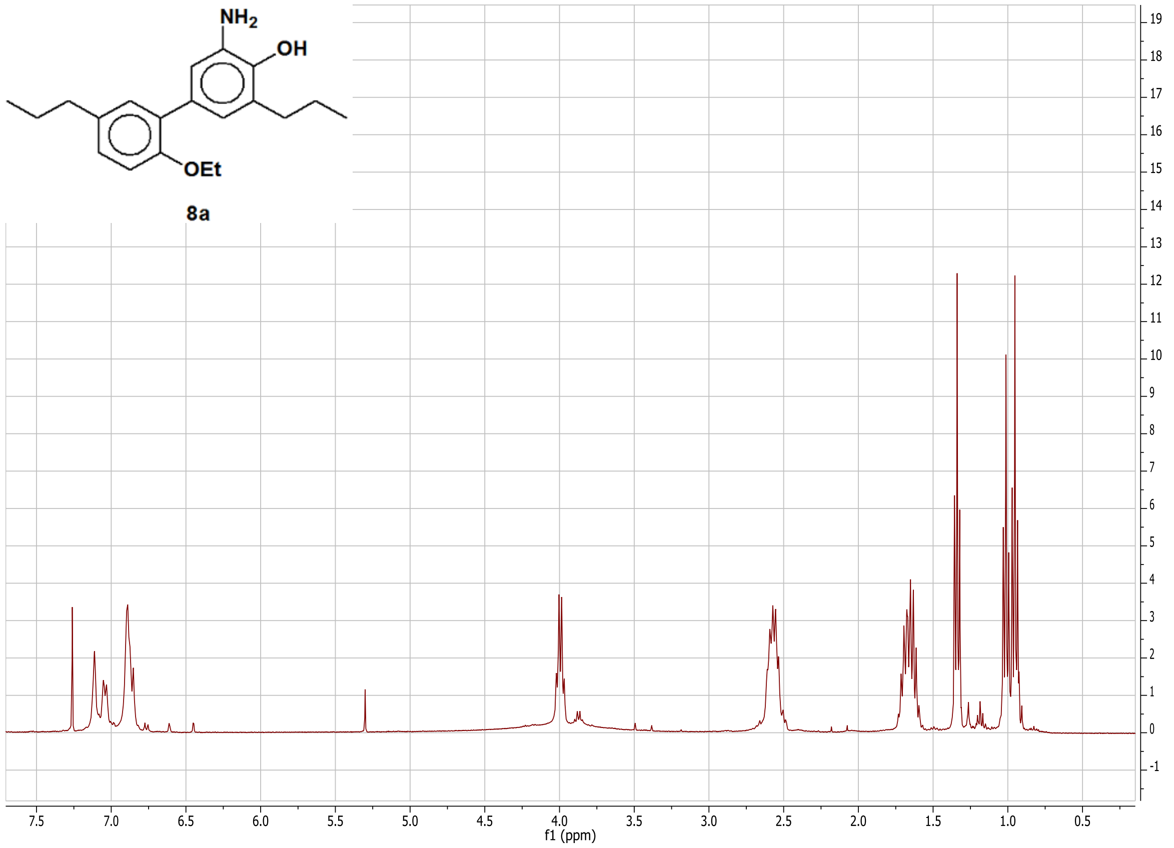

Figure S12. ^{1}H-NMR spectrum ($CDCl_3$, 400 MHz) of **8b**.

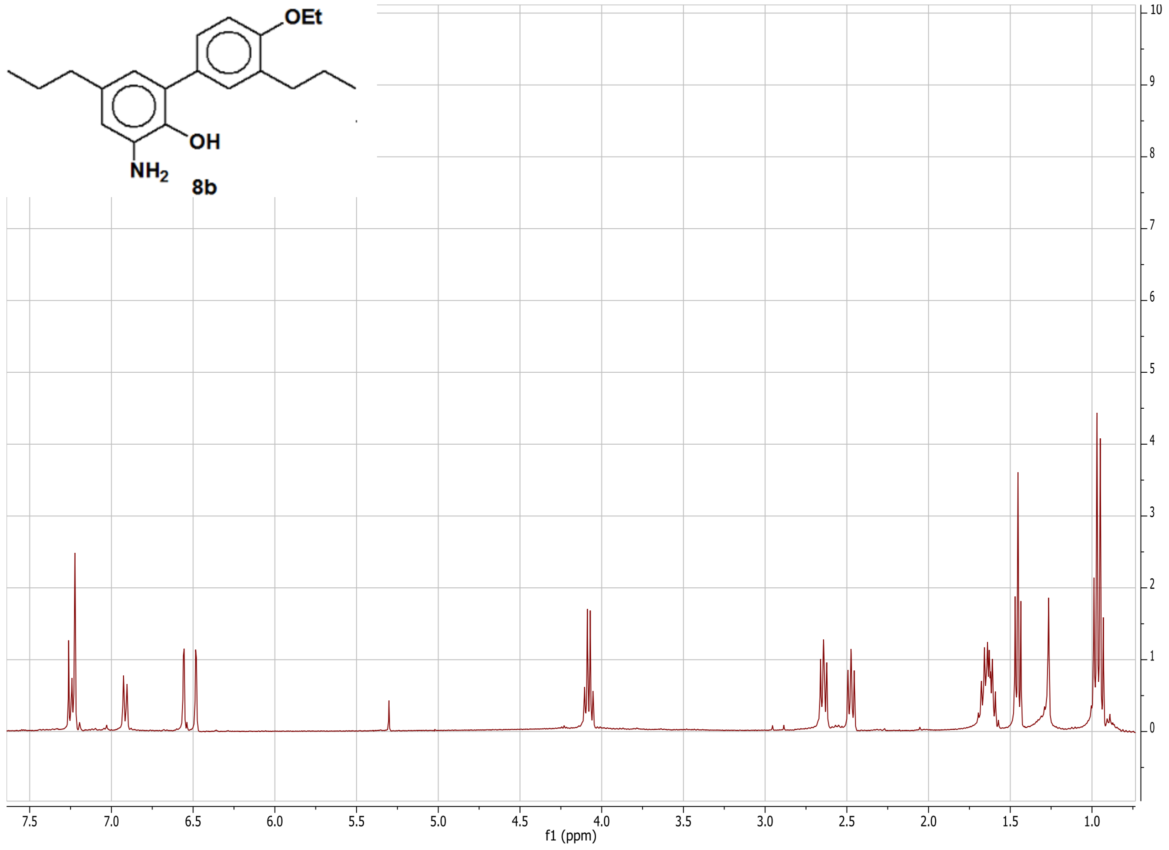

Figure S13. ^{1}H-NMR spectrum ($CDCl_3$, 400 MHz) of **9a**.

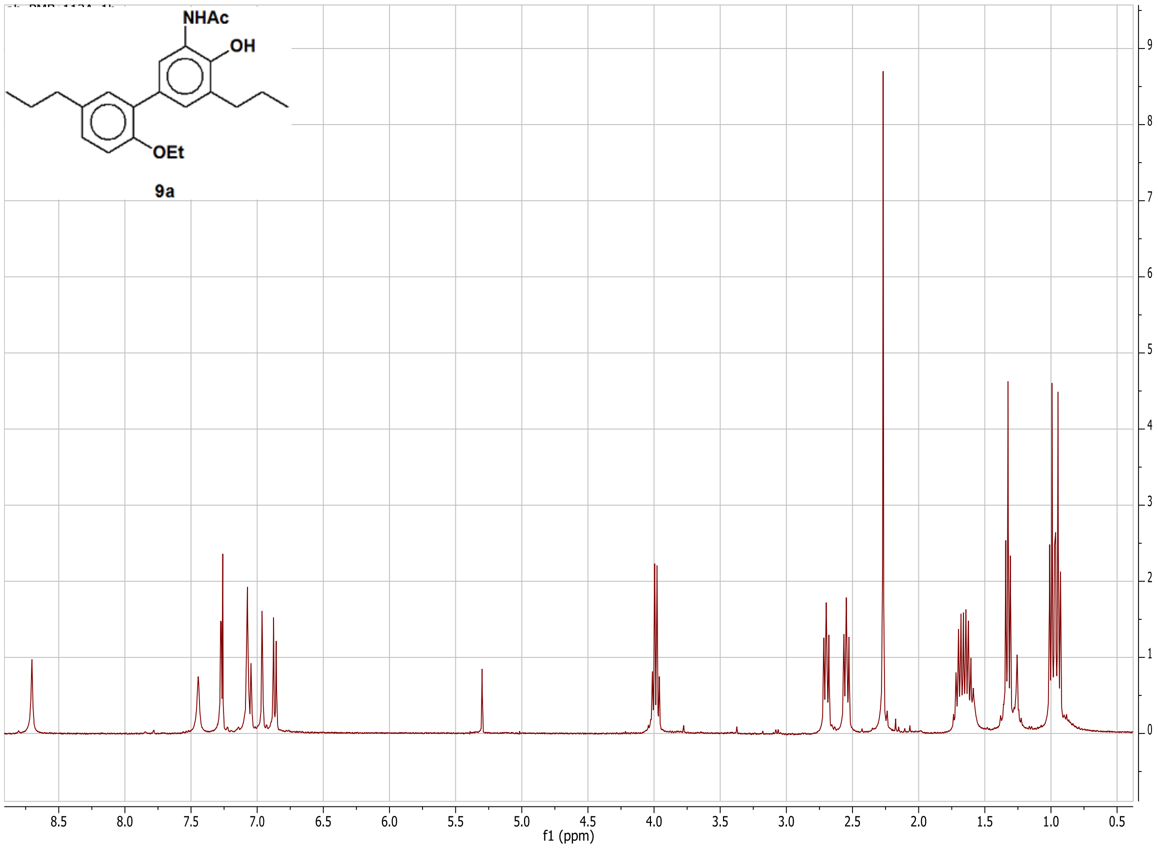

Figure S14. ^{1}H-NMR spectrum ($CDCl_3$, 400 MHz) of **9b**.

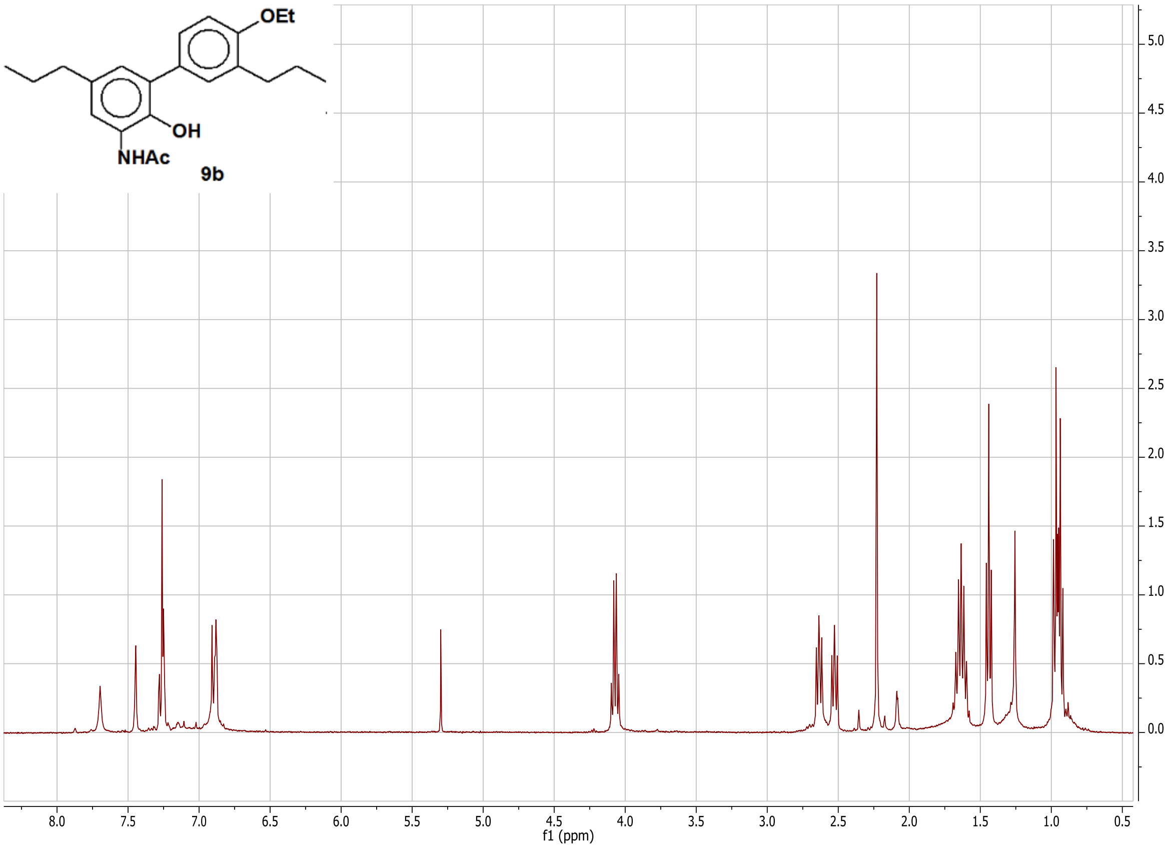

Supplement: Supplementary file 1 [file molecules-19-01223-s001.pdf]
